# Effect of the genetic merit for backfat thickness on the meat quality of steers

**DOI:** 10.1007/s11250-026-05241-0

**Published:** 2026-07-20

**Authors:** Thiago Luís Alves Campos de Araújo, Gelson Luís Dias Feijó, Marina de Nadai Bonin Gomes, Elzania Sales Pereira, Gilberto Romeiro de Oliveira Menezes, Ériklis Nogueira, Luiz Orcírio Fialho de Oliveira, Andrei Pereira Neves, Jaqueline Rodrigues Ferreira, Karla Izidio Latta, Douglas Gomes Vieira, Rodrigo da Costa Gomes

**Affiliations:** 1 Department of Animal Science, Federal University of Ceará, Pici Campus, 810, Fortaleza, CE 60440-900 Brazil; 2 Semi-arid National Institute, Ministry of Science, Technology and Innovation, Av. Francisco Lopes de Almeida, 4000, Campina Grande, PB 58434-700 Brazil; 3 Brazilian Agricultural Research Company (Embrapa), Embrapa Beef Cattle, Radio Maia Av., 830, Campo Grande, MS 79106-550 Brazil; 4 Faculty of Veterinary Medicine and Animal Science, Federal University of Mato Grosso do Sul, Senador Filinto Muller Av., 2443, Campo Grande, MS 79074-460 Brazil; 5 Department of Animal Science, State University of Londrina, Celso Garcia Cid Highway, Km 380, Londrina, PR 86057-970 Brazil

**Keywords:** Aberdeen Angus, Crossbreeding, Expected progeny differences, Fat, Genetic value, Nellore

## Abstract

The objective of the present study was to evaluate the effects of the genetic merit for backfat thickness (GMB) and subcutaneous fat thickness (FT) on meat quality traits of Nellore and crossbred (½ Aberdeen Angus + ½ Nellore) steers. The animals were obtained by mating Nellore cows with Nellore or Aberdeen Angus bulls. Both paternal genetic groups were selected to provide positive (+, high GMB) or negative (-, low GMB) expected progeny differences (EPDs) in terms of backfat thickness. Two hundred and fourteen animals were obtained, reared on a pasture, and finished either in feedlot or semi-confinement systems. The steers were slaughtered, and the backfat visual scores, FT, marbling visual scores and meat color aspects were evaluated for the carcasses (24 h postmortem). In non-aged and aged meat samples (for 14 days), the pH, color, drip loss, cooking loss, and shear force were analyzed. The moisture content and intramuscular fat concentrations of the meat were determined. A second experimental approach was proposed to assess the influence of the FT class (low = 3.66 ± 0.58 mm; medium = 4.99 ± 0.49 mm and high = 6.81 ± 1.06 mm) on the same variables as in the original study. The GMB influenced (*P* < 0.05) the FT and carcass fattening but did not influence (*P* > 0.05) the physicochemical traits or the fat content of the meat. Steers with a low, medium, or high FT provided meats with similar physicochemical attributes. Thus, GMB increases subcutaneous fat deposition without affecting meat quality traits.

**Supplementary Information:**

The online version contains supplementary material available at https://doi.org/10.1007/s11250-026-05241-0.

## Introduction

Knowledge about adipose tissue deposition is important for beef production and quality. Subcutaneous fat deposition is closely associated with carcass and meat quality, mainly because of its thermoinsulating action that prevents *“cold shortening*” (Thompson 2002; Warriss 2010). Owing to its relevance, subcutaneous fat cover has become a criterion for carcass classification and genetic selection and is mainly represented by subcutaneous fat thickness (FT). However, the pursuit to produce carcasses with a higher FT has given rise to paradoxes related to the amount and composition of fat in the carcass (Webb and O’Neill 2008).

Owing to the inconvenience of having excess fat on the carcass, adipose tissue needs to be trimmed during the standardization of cuts, which requires additional labor and reduces commercial yield, impacting the production efficiency (Kelly et al. 2019; Kenny et al. 2020). In addition, compared with that of other body tissues, adipocyte hypertrophy has a high energy cost (Bonnet et al. 2010). This contributes to increased net energy requirements for gain and reduced feed efficiency (Marcondes et al. 2016), which are more obvious as the animal matures (NRC, 2016). Thus, it can be inferred that the excessive deposition of fat in the carcass negatively impacts the production efficiency through a reduction in the feed efficiency and commercial yield.

For these reasons, the meat industry in several countries delimits ideal margins of the FT in the carcass according to market demands. In Brazil, for example, the margin is 3 to 10 mm (Gomes et al. 2021); in Canada, it is between 2 and 7 mm (Bonny et al. 2017); in South Africa, it is between 3 and 7 mm (Bonny et al. 2017); and in the USA, it is between 2.5 and 22.5 mm, with adjustments depending on the carcass (Hale et al. 2013). These criteria encourage the use of tools that predict and control subcutaneous fat deposition in a search for production efficiency.

Advances in the genetic improvement of beef cattle have leveraged the sector and generated increasingly accurate predictions of expected progeny differences (EPDs). This information has enabled the selection of animals with superior productive traits through the genetic merit of their parents (Snijders et al. 2001; Ferraz et al. 2018; de Araújo et al. 2022). In this sense, the genetic merit for backfat thickness (GMB) can aid in the standardization of fattening in bovine carcasses, contributing to production strategies (de Araújo et al. 2022).

Information on the genetic value of available genotypes can help the productive sector according to the needs of each region. In Brazil, the beef cattle industry has joined forces to increase the fattening of the carcasses produced (Gomes et al. 2021), especially in the Nellore breed. On the other hand, the American market has sought to maintain or reduce the FT of carcasses and increase the amount and distribution of marbling (Gonzalez and Phelps 2018). In this context, obtaining divergent phenotypes through genetic selection is advantageous; however, it is important to know the extent of these effects on the quality of products from different genotypes.

The objective of this study was to evaluate the effect of the GMB on meat quality of Nellore and crossbred (½ Aberdeen Angus + ½ Nellore) steers. Our study contemplated an approach using genetically and phenotypically divergent populations for the FT, which should further legitimize the effects of this factor on meat quality. As a result, the effects of the FT class on meat quality of Nellore and crossbred (½ Aberdeen Angus + ½ Nellore) steers were also evaluated to test the hypothesis that the physicochemical attributes of meat from animals classified as having a high FT were different from those of meat from animals classified as having a medium or low FT.

## Materials and methods

### Experimental animals and treatments

The experimental animals were obtained by mating Nellore (N) cows with Nellore (N) or Aberdeen Angus (A) bulls. The two paternal genetic groups were selected for the prediction of expected progeny differences, namely, positive (+, high GMB) or negative (-, low GMB) for backfat thickness (EPDbft). The criteria and names of the bulls chosen to obtain the experimental animals are described in Table 1. A total of 214 animals from the matings described were used as follows: Nellore high GMB (N_N+) = 48, Nellore low GMB (N_N-) = 53, ½ Aberdeen Angus + ½ Nellore high GMB (A_N+) = 51, and ½ Aberdeen Angus + ½ Nellore low GMB (A_N-) = 62, with three experiments (I, II, and III) conducted in different periods and in different systems.

**Table 1:** Criteria used for the selection of bulls and information on the selected bulls used for the production of the animals studied

Product^1^	%Year^3^	Acc^4^	%Bft^5^	Acc^4^	Summary^2^	
N+	< 30%	> 30%	< 5%	> 30%	ANCP (2014)	
N-	< 30%	> 30%	99%	> 30%	ANCP (2014)	
A+	< 30%	> 30%	< 10%	> 30%	AAA (2014)	
A-	< 30%	> 30%	> 65%	> 30%	AAA (2014)	
**Bull name**	**%Year** ^**3**^	**Acc** ^**4**^	**%Bft** ^**5**^	**EPDbft** ^**6**^	**Acc** ^**4**^	**NOS** ^**7**^
N+						48
REM Quilano	4	0.93	0.1	1.09	0.88	9
Relevo do Golias	25	0.82	1	0.50	0.73	7
Quarto JHV	4	0.87	2	0.44	0.77	14
Maximus da Matinha	2	0.79	2	0.36	0.74	9
Samaritano JHV	3	0.84	2	0.38	0.73	9
N-						53
Golias FIV Col	7	0.71	99	-0.54	0.68	12
Nougan do API	6	0.77	99	-0.37	0.68	8
Faracatu JB da Gur	1	0.78	99	-0.33	0.64	11
Imperioso Col	3	0.74	99	-0.22	0.66	15
Donato de Navirai CSCC	3	0.93	99	-0.28	0.79	7
A+						51
Sitz Gunslinger 11612	3	0.36	3	2.21	0.33	15
EF Complement 8088	5	0.82	1	2.13	0.64	8
Sitz RLS Undertaker 11582	5	0.36	3	1.98	0.34	8
Connealy Final Product	5	0.93	2	1.93	0.68	8
SAV Plainsman 0468	15	0.33	2	1.91	0.33	12
A-						62
Connealy Lead On	30	0.96	99	-2.34	0.72	4
SAV Blacksmith 2815	3	0.38	97	-1.30	0.34	16
Quaker Hill Rampage 0A36	1	0.34	95	-1.14	0.39	11
HA PROGRAM 5652	17	0.91	95	-1.14	0.66	19
Circle A Incentive	10	0.67	85	-0.64	0.70	12
Total						214

^1^ Product: N + = Nellore bull with a high genetic merit for backfat thickness; N- = Nellore bull with a low genetic merit for backfat thickness; A+ = Aberdeen Angus bull with a high genetic merit for backfat thickness; A- = Aberdeen Angus bull with a low genetic merit for backfat thickness; ^2^ Summary: Information was obtained from the bull summaries of the National Association of Breeders and Researchers and the American Angus Association (ANCP 2014; AAA 2014); ^3^%Year = Percentile of bulls for yearling weight mean; ^4^ Acc = Accuracy of the trait; ^5^%Bft = Percentile of bulls for subcutaneous fat thickness; ^6^ EPDbft = Expected progeny difference for backfat thickness (in millimeters); ^7^ NOS = Number of offspring used in the study

Experiment I was conducted with calves born in 2016 (*n* = 68) in a pasture rearing system and was finished with keeping castrated steers under semiconfined conditions. Experiment II was conducted with calves born in 2017 (*n* = 70) in a pasture rearing system and was finished under semi-confinement conditions using castrated steers. Experiment III was carried out with calves born in 2017 (*n* = 76) in a pasture rearing system; however, it was finished in feedlot using non-castrated steers.

The rearing systems used were similar for the animals in all three experiments; from calving to weaning, the progenies were kept with the cows in *Brachiaria* spp. pastures and received mineral supplementation *ad libitum*. The calves were weaned by separation from the cows at 8.60 ± 0.55 months of age and had a body weight (BW) of 222.43 ± 28.83 kg (adjusted for 240 days).

### Rearing and finishing phases

All the experiments were carried out by keeping the animals in a single lot on a *Brachiaria* spp. grass pasture under continuous stocking. The steers received a protein-energy supplementation (Appendix A), which was provided at a level of 0.3% of the average BW of the herd, and had free access to water. Every 45 days, the BW of the steers was measured, and they finished the rearing phase with average BWs of 309.51 and 382.27 kg for the N_N and A_N animals, respectively. During this period, the average daily weight gain (ADG) was 0.45 kg/day for the N_N steers and 0.56 kg/day for the A_N steers.

Afterward, the animals in Experiments I and II were surgically castrated through a lateral incision (Silva et al. 2009) and kept under semiconfined conditions. The steers remained in pastures formed with *Brachiaria brizantha* cv. Xaraés, and their diet was supplemented with a concentrated feed (Appendix B) offered daily at the level of 1.5% of the herd average BW.

Animals in Experiment III remained non-castrated at the end of the rearing stage and were finished in a feedlot and received a total mix ration (TMR) twice a day. The TMR contained 50% of sorghum silage and 50% of a dry matter-based concentrated feed. The formulation and composition details of the TMR are available in Appendix C.

At the end of the finishing phase, the steers were 26.24 ± 2.36 months old on average. During this period, the ADG of the N_N animals was 0.98 kg/day, and the final BW was 539.70 kg; the ADG of the A_N animals was 1.20 kg/day, and the final BW was 592.33 kg.

### Slaughter and 24 h postmortem evaluations

The animals were slaughtered following humane procedures regulated by Brazilian legislation (RIISPOA, 2017). The carcasses were individually identified, longitudinally sectioned, weighed and cooled at 2 °C for 24 h. After the cooling period, the backfat visual score was evaluated using a scale from 1 to 5, with scores assigned in relation to the amount of carcass subcutaneous fat, as recommended by Gomes et al. (2021). Next, the longissimus thoracis (LT) muscle was sectioned between the 12th and 13th ribs, making it possible to measure the subcutaneous fat thickness (FT) with a digital caliper. The marbling score was calculated using the standard classification proposed by the USDA (2017). For each degree of marbling, the following numerical values were assigned in ascending order, including intermediate values: traces (tr- = 1, tr0 = 2 and tr + = 3); slight (sl- = 4, sl0 = 5 and sl + = 6); small (sm- = 7, sm0 = 8 and sm + = 9); modest (mo- = 10, mo0 = 11 and mo + = 12); moderate (md- = 13, md0 = 14 and md + = 15); and abundant (ab- = 16, ab0 = 17 and ab + = 18).

Subsequently, the LT portion between the 10th and 12th ribs of each carcass were removed. After a blooming period (~ 30 min), the instrumental color aspects of the meat, cleaned and unstained surfaces of the subcutaneous fat on the LT muscle were evaluated. A MiniScan XE Plus colorimeter (HunterLab, Reston, USA) was used, with a 10° iris opening angle and a D65 illuminant (similar to sunlight, including the ultraviolet region), programmed to measure the following parameters of the CIELab system: luminosity (L* 24 h postmortem) and intensities of red (a* 24 h postmortem) and yellow (b* 24 h postmortem). The means resulting from the measurements taken at three points in the samples were recorded.

### Meat quality analysis

The LT muscle was used for meat quality evaluations. Three 2.5-cm thick steaks (I, II and III) were removed in the same sequence and anatomical position in the caudal-cranial direction. Steak I of each sample was aged for fourteen days at 4 °C before being frozen (steak I – aged), while the other two steaks (steak II and steak III – non-aged) were vacuum packed and stored at -20 °C as soon as they were removed from the samples.

One of the subsamples of the non-aged meat (steak III) was ground in a multiprocessor and subjected to freeze-drying for 38 h in an LJJ freeze-dryer (JJ Científica, São Carlos, SP, Brazil). The moisture amount removed during freeze-drying was determined by the difference in the weights of the sample before and after the process. Subsequently, the total moisture content was determined using method 967.03 of AOAC (2016). In lyophilized samples, the total fat content was determined according to the AM 5 − 04 procedure established by AOCS (2017) using the ANKOM XT15 system (ANKOM Technology, Macedonia, USA) and petroleum ether as the solvent.

Other analyses were carried out in both steaks I – matured, and steaks II – unmatured, following the guidelines of AMSA (2016). For this purpose, the subsamples were thawed under refrigeration (4 °C), removed from the packages, and placed on cooking grids. The mass of the exudate contained in the package was carefully weighed on an LS2 precision scale (Marte Científica, MG, Brazil) to obtain the drip losses. Subsequently, the color aspects (L*, a*, and b*) were measured in each subsample as previously described.

The pH of the meat was measured using an HI99163 potentiometer (Hanna Instruments, Woonsocket, USA). After the weight was recorded, each sample was cooked in an LTedesco 6GNs electric oven (Tedesco Stoves, Caxias do Sul, RS, Brazil) at 190 °C with forced air circulation until the temperature in the geometric center of the subsample, which was individually measured using a digital thermocouple, reached 71 °C. The weight loss was calculated by the weight difference before and after cooking.

With the aid of a corer, six cylindrical subsamples with a diameter of 1.27 cm were removed to determine the shear force (SF) parallel to the direction of the muscle fibers of each cooked subsample (steaks I and II). Each of the cylinders was sheared on a 235 6X analog texturometer (Salter Brecknell, Birmingham, England) with a 1.18-mm thick Warner–Bratzler blade. The force required for perpendicular rupture of the cylinders was recorded. The mean of the six measurements performed to obtain the SF value was subsequently calculated. The tenderization promoted by aged meat was calculated by the difference between the SFs of aged (steak I) and non-aged (steak II) samples.

A second experimental approach was proposed to evaluate the effects of the FT class on the same parameters as those in the original study. After slaughter and evaluation of the FT in the progenies, the steers in each experiment and breed were classified into three classes according to their individual FT values, regardless of their paternal EPD for this trait. Animals with an FT above 0.5 standard deviations (FT > 0.5 σ, *n* = 82) in relation to the general mean of each breed and system were considered to be in the high class; individuals with the FT between − 0.5 and 0.5 standard deviations (-0.5 σ < FT < 0.5 σ, *n* = 66) were considered to be in the middle class; and animals with an FT lower than − 0.5 σ (FT < -0.5 σ, *n* = 62).

### Statistical analysis

A randomized block design was used, with a factorial arrangement of 2 × 2 (paternal breeds × GMB) and 2 × 3 (paternal breeds × FT classes) according to the following mathematical models:

$$\begin{aligned} & {Y}_{ijlmn}=\mu+{P}_{i}+{G}_{j}+{L}_{l}+{E}_{m}+{\left(P\times G\right)}_{ij}+{\epsilon}_{ijlmn}\text{ and}\\&{Y}_{iklmn}=\mu+P_{i}+{C}_{k}+{L}_{l}+{E}_{m}+{\left(P\times C\right)}_{ik}+{\epsilon}_{iklmn},\end{aligned}$$

Where: Yijlmn = dependent variable observed in animal “n”, paternal breed “i”, GMB “j”, lot “l” and experiment “m”; Y_iklmn_ = dependent variable observed in animal “n”, paternal breed “i”, FT class “k”, lot “l” and experiment “m”; µ = population mean or global constant; Pi = effect of paternal breed “i”; G_j_ = effect of GMB “j”; C_k_ = effect of FT class “k”; L_l_ = effect of lot “l”; E_m_ = effect of experiment “m”; (P x G)_ij_ = interaction between paternal breed “i” and GMB “j”; (P x C)_ik_ = interaction between paternal breed “i” and FT class “k”; and ɛ_ijlmn_ and ɛ_iklmn_ = random error not observed.

The data were subjected to restricted maximum likelihood analysis using the PROC MIXED procedure of SAS On Demand for Academics, considering the lot and experiment as random effects. The random lot effect included individual variables such as age and weight at slaughter, while the random experiment effect included variations in origin, year of birth and finishing system. Because there were more than two levels, the Tukey–Kramer test was applied between the means of the FT classes. A significance level of 5% was used.

## Results

### Effects of the GMB and paternal breed on meat quality

The paternal mean EPDbft, as well as the FT and backfat visual scores of the animals, are shown in Table 2. There was no effect of GMB, nor an interaction effect (*P* > 0.05) between the paternal breed and GMB on the variables evaluated 24 h *postmortem* (Table 2). Thus, animals with high and low GMB provided carcasses and meat with similar marbling scores and color aspects, regardless of the paternal breed (Nellore or Aberdeen Angus). There was an effect (*P* < 0.05) of the paternal breed for most of the 24-h *postmortem* evaluations, except for b* of meat and L* of subcutaneous fat. The Nellore animals produced meat with lower marbling scores, a higher luminosity (L*) and a lower red color intensity (a*), while the A_N steers produced subcutaneous fat with greater red (a*) and yellow (b*) intensities.

**Table 2:** Effects of paternal breed and genetic merit for backfat thickness on marbling and the color of fresh meat (24 h *postmortem*) and subcutaneous fat of steers

Variable	Paternal^1^			GMB^2^			SEM^3^	*P*-Value			
	Angus (*n* = 113)	Nellore (*n* = 101)		Low (*n* = 115)	High (*n* = 99)			Paternal^1^		GMB^2^	Interaction
EPD^4^ backfat thickness (mm)	-	-		-0.79	1.32		0.08	-		< 0.0001	< 0.0001
Backfat thickness (mm)	5.26	4.64		4.49	5.40		0.11	0.0071		< 0.0001	0.3893
Backfat visual score	2.55	2.58		2.44	2.69		0.04	0.6392		0.0002	0.1276
Marbling score	6.07	4.69		5.48	5.28		0.16	< 0.0001		0.4714	0.9348
L* fresh meat (%)	34.63	35.89		35.02	35.50		0.16	< 0.0001		0.0952	0.1689
a* fresh meat (%)	19.80	18.79		19.27	19.32		0.16	0.0002		0.8197	0.4612
b* fresh meat (%)	15.55	15.38		15.42	15.52		0.14	0.5128		0.6577	0.9454
L* subcutaneous fat (%)	73.90	74.71		74.05	74.26		0.21	0.2525		0.5960	0.6171
a* subcutaneous fat (%)	9.92	8.94		9.28	9.57		0.15	0.0029		0.3283	0.4522
b* subcutaneous fat (%)	22.34	20.93		21.56	21.71		0.23	0.0008		0.6695	0.8265
**Factors interaction**	**Angus**					**Nellore**					
	**Low**		**High**			**Low**			**High**		
EPD^4^ backfat thickness (mm)	-1.17^d^		2.05^a^			-0.35^c^			0.54^b^		

^1^ Paternal breed of progenies; ^2^ Genetic merit for backfat thickness; ^3^ Standard error of the mean; ^4^ Expected difference in progeny; L* = Luminosity. a* = red color intensity. b* = yellow color intensity; ^abc^ Means with distinct letters on the same lines differ by the Tukey–Kramer test at 5% significance

There was an interaction effect (*P* < 0.05) between the paternal breed and GMB on the aspects of the color of the matured meat (Table 3). The L* and b* values of the N_N animals differed from those of the A_N animals for the matured meat, with the first genetic group showing greater averages for these traits. There was no effect (*P* > 0.05) of the GMB on the instrumental quality or fat and moisture contents of the meat. There was an effect (*P* < 0.05) of the paternal breed on most of the instrumental quality variables and on the fat content of the meat.

**Table 3:** Effects of paternal breed and genetic merit for backfat thickness on meat quality (*longissimus thoracis*) of steers

Variable	Paternal^1^		GMB^2^			SEM^3^	*P*-Value			
	Angus (*n* = 113)	Nellore (*n* = 101)	Low (*n* = 115)	High (*n* = 99)			Paternal^1^		GMB^2^	Interaction
pH	5.61	5.60	5.60	5.60		0.01	0.4615		0.9078	0.8851
L* (%)	35.65	37.19	36.40	36.44		0.17	< 0.0001		0.9202	0.2857
a* (%)	12.93	13.07	13.18	12.82		0.13	0.5362		0.0886	0.2145
b* (%)	12.07	12.58	12.41	12.25		0.08	0.0019		0.3039	0.1805
Drip loss (%)	8.53	9.44	9.06	8.97		0.30	0.1538		0.8515	0.8382
Cooking loss (%)	23.21	24.35	23.79	23.77		0.34	0.0749		0.9832	0.7342
SF^4^ (N)	54.61	71.91	62.89	63.62		1.40	< 0.0001		0.7525	0.9113
Moisture (g/100 g meat)	81.08	80.89	80.95	81.02		0.40	0.8169		0.9182	0.2307
Fat (g/100 g meat)	1.40	1.13	1.32	1.22		0.04	0.0045		0.2314	0.6393
Aged meat^5^										
pH	5.70	5.67	5.69	5.68		0.01	0.1035		0.8523	0.4032
L* (%)	37.36	39.21	38.24	38.34		0.17	< 0.0001		0.7404	0.0061
a* (%)	12.09	12.28	12.18	12.19		0.13	0.4287		0.9380	0.2460
b* (%)	12.22	12.92	12.58	12.56		0.08	< 0.0001		0.8679	0.0024
Drip loss (%)	9.54	10.17	9.60	10.11		0.25	0.1936		0.2358	0.5956
Cooking loss (%)	23.67	23.70	23.80	23.57		0.34	0.9637		0.6583	0.8783
SF^4^ (N)	35.71	48.32	40.61	43.41		1.02	< 0.0001		0.1004	0.8866
Tenderization after aging^5^ (N)	-17.87	-25.04	-21.30	-21.61		1.11	0.0018		0.8786	0.2711
Tenderization after aging ^5^ (%)	-32.06	-31.90	-32.55	-31.40		1.26	0.9533		0.6191	0.2561
**Factors interaction**	**Angus**				**Nellore**					
	**Low**		**High**		**Low**			**High**		
L* of aged meat^5^ (%)	36.90^c^		37.81^bc^		39.58^a^			38.86^ab^		
b* of aged meat^5^ (%)	12.03^c^		12.41^bc^		13.14^a^			12.71^ab^		

^1^ Paternal breed of progenies; ^2^ Genetic merit for backfat thickness; ^3^ Standard error of the mean; ^4^ Shear force; ^5^ Aging for 14 days; ^abc^ Means with distinct letters on the same lines differ by the Tukey–Kramer test at 5% significance

Regarding the instrumental aspects of color, the N_N animals provided meat with higher luminosity (L*) and yellow intensity (b*) than did the A_N steers, both in the matured and unmatured meat (Table 3). In the same way, regardless of the aging process, the meat of the A_N animals lost more weight during cooking; however, the SF of the meat of these animals was lower. With aging, the SF of the N_N meat decreased more than that of the A_N steer meat. While the SF of the A_N meat decreased by 12.6 Newtons after aged, that of the N_N meat decreased by 17.30 Newtons. However, the same result was not obtained for percentage decrease (%) of the shear force before and after aged.

### Effect of the subcutaneous fat thickness class on meat quality

The paternal means of EPDbft, as well as the FT and the backfat visual scores of the animals, are presented in Table 4. As recommended, the FT and the backfat visual scores that differed (*P* < 0.05) between the FT classes and effects of the paternal breed, as observed in Study I, were repeated in Study II.

**Table 4:** Effects of the paternal breed and subcutaneous fat thickness (FT) class^1^ on marbling and the color of fresh meat (24 h postmortem) and subcutaneous fat of steers

Variable	Paternal^2^		FT class^1^			SEM^3^	*P*-Value		
	Angus (*n* = 113)	Nellore (*n* = 101)	Low (*n* = 82)	Medium (*n* = 66)	High (*n* = 62)		Paternal^2^	FT^1^	Interaction
EPD^4^ backfat thickness (mm)	0.34	0.08	-0.07^c^	0.18^b^	0.51^a^	0.09	0.1508	0.0214	0.3322
Backfat thickness (mm)	5.38	4.93	3.66^c^	4.99^b^	6.81^a^	0.11	0.0003	< 0.0001	0.7237
Backfat visual score	2.51	2.62	2.40^b^	2.50^b^	2.81^a^	0.03	0.1207	< 0.0001	0.0321
Marbling score	6.11	4.75	5.04	5.58	5.68	0.16	< 0.0001	0.1117	0.5761
L* fresh meat (%)	34.59	35.86	35.13	34.91	35.64	0.16	0.0002	0.1386	0.4712
a* fresh meat (%)	19.82	18.78	19.10^b^	19.09^b^	19.73^a^	0.16	0.0002	0.0471	0.4605
b* fresh meat (%)	15.46	15.39	15.18^b^	15.24^b^	15.85^a^	0.13	0.8111	0.0269	0.4249
L* subcutaneous fat (%)	73.85	74.46	73.98	74.10	74.40	0.21	0.1800	0.6774	0.8827
a* subcutaneous fat (%)	9.92	8.91	9.26	9.52	9.45	0.15	0.0022	0.7454	0.9159
b* subcutaneous fat (%)	22.44	20.86	21.60	21.65	21.69	0.23	0.0002	0.9748	0.7665

^1^ High = FT > 0.5σ for each paternal breed. Medium = -0.5σ < FT < 0.5σ for each paternal breed. and Low = FT < -0.5σ for each paternal breed; ^2^ Paternal breed of progenies; ^3^ Standard error of the mean; ^4^ Expected progeny difference; ^abc^ Means with distinct letters on the same lines differ by the Tukey–Kramer test at 5% significance

There was no interaction effect (*P* > 0.05) between the paternal breed and FT class on the variables evaluated 24 h *postmortem* (fresh meat - Table 4). Similarly, there was no effect (*P* > 0.05) of the FT class on marbling or on instrumental color attributes evaluated in fresh meat, except for b*. Yellow color intensity was greater in the fresh meat of animals with a high FT than in those with a low FT, while this parameter in animals with a medium FT did not differ from that of the others.

There was no interaction effect (*P* > 0.05) between the paternal breed and FT class on the physicochemical attributes of the matured and non-aged meat (Table 5). Except for the intramuscular fat content, the FT class did not influence the physicochemical attributes of the meat quality. The intramuscular fat content of the meat of the animals classified as having a high FT was greater than that of the other groups (classified as having a medium or low FT), and the two groups of meat did not differ from each other.

**Table 5:** Effects of the paternal breed and subcutaneous fat thickness (FT) class^1^ on the meat quality (*longissimus thoracis*) of steers

Variable	Paternal^2^		FT class^1^			SEM^3^	*P*-Value		
	Angus (*n* = 113)	Nellore (*n* = 101)	Low (*n* = 82)	Medium (*n* = 66)	High (*n* = 62)		Paternal^2^	FT^1^	Interaction
pH	5.62	5.59	5.61	5.61	5.60	0.01	0.2535	0.8022	0.4415
L* (%)	35.62	37.21	36.28	36.38	36.58	0.17	< 0.0001	0.7376	0.6898
a* (%)	12.96	13.14	12.77	13.17	13.22	0.14	0.4610	0.1420	0.9316
b* (%)	12.09	12.61	12.19	12.29	12.58	0.08	0.0020	0.1174	0.7019
Drip loss (%)	8.70	9.47	8.95	9.30	9.01	0.30	0.2004	0.8448	0.6286
Cooking loss (%)	23.22	24.26	23.88	22.96	24.37	0.34	0.1084	0.1707	0.1588
SF^4^ (N)	55.21	71.15	64.03	62.51	62.99	1.42	< 0.0001	0.8486	0.2916
Moisture (g/100 g meat)	81.13	80.90	81.69	81.30	80.07	0.41	0.7870	0.1845	0.5548
Fat (g/100 g meat)	1.39	1.14	1.17^b^	1.19^b^	1.44^a^	0.04	0.0075	0.0130	0.2489
Aged meat^4^									
pH	5.62	5.59	5.61	5.61	5.60	0.01	0.2535	0.8022	0.4415
L* (%)	35.62	37.21	36.28	36.38	36.58	0.17	< 0.0001	0.7376	0.6898
a* (%)	12.96	13.14	12.77	13.17	13.22	0.14	0.4610	0.1420	0.9316
b* (%)	12.09	12.61	12.19	12.29	12.58	0.08	0.0020	0.1174	0.7019
Drip loss (%)	9.54	10.31	9.80	9.98	10.00	0.25	0.1284	0.9025	0.6493
Cooking loss (%)	23.65	23.62	23.86	23.44	23.62	0.34	0.9619	0.7933	0.9387
SF^4^ (N)	35.57	48.22	42.15	41.24	42.29	1.03	< 0.0001	0.8702	0.2843
Tenderization after aging ^5^ (N)	-32.67	-32.11	-32.55	-31.22	-33.39	1.28	0.8356	0.7583	0.1356

^1^ High = FT > 0.5σ for each paternal breed. Medium = -0.5σ < FT < 0.5σ for each paternal breed. and Low = FT < -0.5σ for each paternal breed; ^2^ Paternal breed of progenies; ^3^ Standard error of the mean; ^4^ Aging for 14 days; ^abc^ Means with distinct letters on the same lines differ by the Tukey–Kramer test at 5% significance

## Discussion

### Effects of the GMB and paternal breed on meat quality

The effects and interactions between the factors for these variables were discussed in a previous publication (de Araújo et al. 2022). The results showed that there were few and inconclusive interactions between the GMB and paternal breed factors on the physicochemical attributes of meat. As a result, it can be inferred that the factors studied are independent in relation to most of the variables analyzed.

Our research revealed that animals with divergent GMB expressed this trait in their phenotypes, which resulted in individuals with distinct FT and backfat score, indicating that there is a distinction in lipid metabolism between these animals (de Araújo et al. 2022). However, even the animals with low GMB did not present with FT values lower than the minimum of 3 mm, as recommended to avoid damage caused by rapid cooling (Prado et al. 2008), which may explain the absence of an effect of the GMB on the physicochemical attributes of the meat.

The absence of an effect of the GMB on the marbling score and fat content of the meat indicates that the genetic mechanism that modulates lipid metabolism in subcutaneous adipose tissue (SAT) does not influence the rate of fat deposition in intramuscular adipose tissue (IAT). It is well documented that simple genetic correlations are low between FT and marbling, parameters related to fat deposition in SAT and IAT, respectively (Bertrand et al. 2001; Londoño-Gil et al. 2022). The divergent biochemical mechanisms between these tissues are evident, with an emphasis on the primary substrates for lipogenesis, namely, glucose in IAT and acetate in SAT (Smith and Crouse 1984; Rhoades et al. 2007). However, genetic mechanisms that regulate adipogenesis and the interdependencies between different tissues are still being elucidated (Urrutia et al. 2020).

When analyzing RNA sequencing data from the IAT, SAT, and perirenal adipose tissue of cattle, Sheng et al. (2014) revealed that there was a pattern involving 110 differentially expressed genes between the tissues. The authors also observed a greater occurrence of alternative splicing events in SAT, which denotes a greater dynamism in the translation of genetic information into biological functions. Hudson et al. (2020) reinforced the wide divergence of gene expression between the SAT and IAT and highlighted that the main differences were related to the metabolism of carbohydrates, cholesterol, and retinoic acid. This evidence may be key to explaining how the GMB modulates lipid metabolism only in SAT. However, in our study, the gene expression parameters necessary to prove these hypotheses were not evaluated.

The results observed among the studied breeds have been widely described in the literature, indicating that crossbred animals (1/2 Aberdeen Angus + 1/2 Nellore, A_N) provide meats with greater marbling and intramuscular fat (Oliveira et al. 2011; Chardulo et al. 2013), greater cooking loss (Oliveira et al. 2011), a lower shear force (Oliveira et al. 2011; Miguel et al. 2014; Aroeira et al. 2017; Barcellos et al. 2017), and lower luminosity and yellow intensity (Miguel et al. 2014; Aroeira et al. 2017) than do Nellore animals. Our study reinforces the benefits of use of Aberdeen Angus as a paternal breed in adopted production systems upon physicochemical parameters for meat quality. In addition, such benefits can be observed even in a population of individuals selected for divergent fattening carcass traits.

### Effects of subcutaneous fat thickness class on meat quality

Several studies, including a meta-analysis (Boito et al. 2017), empirical studies (Pflanzer and de Felício 2011; Maggioni et al. 2012), and a multivariate principal component analysis (Baldassini et al. 2017), have evaluated the impact of FT on beef meat quality. In the present research, we evaluated the effects of FT on the qualitative attributes of meat via controlled genetic, environmental and maturity factor experiments, with a large and comprehensive sample population.

The greater a* and b* color intensities in fresh meat (24 h *postmortem*) of the animals with a high FT could be related to a slight variation in the internal temperature of the muscle during cooling in response to the thermal insulation effect obtained with the higher backfat in the carcass of these animals (MacDougall 1982). *Rigor mortis* settles more rapidly at higher chamber temperatures (Young et al. 1999), and during this process, the myofibrils become more reflective (MacDougall 1982). However, the color attributes measured during a short postslaughter period are not definitive parameters of the color appearance and have a low correlation with the meat color in the following days or weeks (Young et al. 1999); this was evidenced by the similarity of the color attributes of the unmatured and matured meat between the FT classes.

The higher intramuscular fat content in the meat of steers with a high FT observed in this study is an indication that these animals could be in transition to an increase in adipocyte differentiation in the IAT. According to Du et al. (2013), with the development of the animal, preadipocytes present in various body tissues mature to adipocytes by hyperplasia, and the IAT is the last in the order of priority of differentiation. However, greater fat deposition was not visually perceptible since the marbling score was not influenced. It is important to emphasize that the progenies were slaughtered relatively young (26.24 ± 2.36 months, 0 to 2 permanent teeth), and these effects could be more obvious with the advance of maturity in these animals.

This study reinforces that an FT above the minimum value (2 to 3 mm) does not greatly influence the physicochemical attributes of meat quality, even in populations that are genetically divergent for subcutaneous fat deposition. Considering the productive and economic impacts of promoting high fat deposition on carcasses, it can be inferred that it would not be necessary to increase the FT much above 2 to 3 mm to improve the physicochemical attributes. In this sense, the increase in the proportion of fat in the carcass at medium and high levels is justified only for meeting the demands for cuts with greater fat thickness, such as those intended for the grilling or dry-heat cooking methods, such as in barbecues.

Animals with divergent GMB values provide meats with similar physicochemical attributes. However, in populations with divergent GMB values, the physicochemical attributes of meat quality are improved when the Aberdeen Angus breed is used for Nellore dams. The FT class did not influence most of the physicochemical attributes of the meat, indicating that it would not be necessary to increase the FT much above 2 to 3 mm for meat protection.

## Supplementary Information

Below is the link to the electronic supplementary material.

Supplementary Material 1

## Data Availability

Data will be made available by the corresponding author upon reasonable request.

## References

[CR1] AMSA (2016) Research guidelines for cookery, sensory evaluation, and instrumental tenderness measurements of meat, 2nd edn. American Meat Science Association, Champaign, IL)

[CR2] AOAC (2016) Official Methods of Analysis, 20th edn. Association of Official Agricultural Chemists, Washington, DC)

[CR3] AOCS (2017) Rapid determination of oil/fat utilizing high-temperature solvent extraction, 7th edn. Urban, IL

[CR4] Aroeira C.N., de Almeida Torres Filho R, Fontes PR, de Lemos Souza Ramos A, de Miranda Gomide LA, Ladeira MM, Ramos EM (2017) Effect of freezing prior to aging on myoglobin redox forms and CIE color of beef from Nellore and Aberdeen Angus cattle. Meat Sci 125:16–21 (Elsevier Ltd) 10.1016/j.meatsci.2016.11.010 27883957

[CR5] Baldassini WA, Chardulo LAL, Silva JAV, Malheiros JM, Dias VAD, Espigolan R, Baldi FS, Albuquerque LG, Fernandes TT, Padilha PM (2017) Meat quality traits of Nellore bulls according to different degrees of backfat thickness: A multivariate approach. Anim Prod Sci 57:363–370

[CR6] Barcellos VC, Mottin C, Passetti RAC, Guerrero A, Eiras CE, Prohmann PEF, Vital ACP, Prado IN, do (2017) Carcass characteristics and sensorial evaluation of meat from Nellore steers and crossbred Angus vs. Nellore bulls Acta Scientiarum Anim Sci 39:437

[CR7] Bertrand JK, Green RD, Herring WO, Moser DW (2001) Genetic evaluation for beef carcass traits. J Anim Sci 79:E190

[CR8] Boito B, Kuss F, de Menezes LFG, Lisbinski E, Paris M (2017) Influence of subcutaneous fat thickness on the carcass characteristics and meat quality of beef cattle Ciência. Rural 48:1CullmannJ.R.

[CR9] Bonnet M, Cassar-Malek I, Chilliard Y, Picard B (2010) Ontogenesis of muscle and adipose tissues and their interactions in ruminants and other species. Animal 4:1093–1109 10.1017/S1751731110000601 22444612

[CR10] Bonny S, Polkinghorne R, Strydom P, Matthews K, López-Campos Ó, Nishimura T, Scollan N, Pethick D, Hocquette J-F (2017) Quality assurance schemes in major beef-producing countries. In: New aspects of meat quality. Elsevier, pp 223–255

[CR11] Chardulo LAL, Silveira AC, Vianello F (2013) Analytical aspects for tropical meat quality assessment. In: Lima GPP, Vianello F (eds) Food quality, safety and technology. Springer Vienna, pp 53–62

[CR12] de Araújo TLAC, Feijó GLD, Neves AP, Nogueira É, de Oliveira LOF, Gomes MDNB, do Egito AA, Ferraz ALJ, Menezes GRDO, Latta KI, Ferreira JR, Vieira DG, Pereira ES, Gomes RDC (2022) Effect of genetic merit for backfat thickness and paternal breed on performance, carcass traits, and gene expression in subcutaneous adipose tissue of feedlot-finished steers. Livest Sci 263

[CR27] de Oliveira IM, Paulino PVR, Marcondes MI, de Valadares Filho S, Cavali J, Prados LF, de Duarte MS, Detmann E (2011) Beef quality traits of Nellore, F1 Simmental × Nellore and F1 Angus × Nellore steers fed at the maintenance level or ad libitum with two concentrate levels in the diet. Rev Bras Zootec 40:2894–2902

[CR13] Du M, Huang Y, Das AK, Yang Q, Duarte MS, Dodson MV, Zhu M-J (2013) Meat science and muscle biology symposium: manipulating mesenchymal progenitor cell differentiation to optimize performance and carcass value of beef cattle. J Anim Sci 91:1419–1427 10.2527/jas.2012-5670 23100595

[CR14] Ferraz MVC, Pires AV, Santos MH, Silva RG, Oliveira GB, Polizel DM, Biehl MV, Sartori R, Nogueira GP (2018) A combination of nutrition and genetics is able to reduce age at puberty in Nelore heifers to below 18 months. Animal 12:569–574. 10.1017/S1751731117002464 29056108

[CR15] Gomes MDNB, Feijó GLD, Duarte MT, da Silva LGP, Surita LMA, Pereira MWF (2021) Manual de avaliação de carcaças bovinas, 1st edn. UFMS, Campo Grande, MS

[CR16] Gonzalez JM, Phelps KJ (2018) United States beef quality as chronicled by the National Beef Quality Audits, Beef Consumer Satisfaction Projects, and National Beef Tenderness Surveys - A review. Asian-Australasian J Anim Sci 31:1036–1042 (Asian-Australasian Association of Animal Production Societies (AAAP)) 10.5713/ajas.18.0199 PMC6039333 29879824

[CR17] Hale DS, Goodson K, Savell JW (2013) USDA beef quality and yield grades. College Station, TX

[CR18] Hudson NJ, Reverter A, Griffiths WJ, Yutuc E, Wang Y, Jeanes A, McWilliam S, Pethick DW, Greenwood PL (2020) Gene expression identifies metabolic and functional differences between intramuscular and subcutaneous adipocytes in cattle. BMC Genomics 21:1–23 (BMC Genomics) 10.1186/s12864-020-6505-4 PMC6986065 31992204

[CR19] Kelly DN, Murphy C, Sleator RD, Judge MM, Conroy SB, Berry DP (2019) Feed efficiency and carcass metrics in growing cattle. J Anim Sci 97:4405–4417 10.1093/jas/skz316 PMC6827399 31593986

[CR20] Kenny D, Murphy CP, Sleator RD, Judge MM, Evans RD, Berry DP (2020) Animal-level factors associated with the achievement of desirable specifications in Irish beef carcasses graded using the EUROP classification system. J Anim Sci 98 10.1093/jas/skaa191 PMC7333216 32516387

[CR21] Londoño-Gil M, Cardona-Cifuentes D, Rodríguez JD, Brunes LC, Magnabosco CU, Pereira ASC, Peripolli E, Lôbo RB, Baldi F (2022) Heritability and genetic correlations between marbling in longissimus dorsi muscle and conventional economic traits in Nellore beef cattle. Trop Anim Health Prod 54:274 (Springer Science and Business Media B.V.) 10.1007/s11250-022-03293-6 36068366

[CR22] MacDougall DB (1982) Changes in the colour and opacity of meat. Food Chem 9:75–88

[CR23] Maggioni D, Prado IN, Zawadzki F, Valero MV, de Marques JA, Bridi AM, Moletta JL, dos Abrahao JJS (2012) Grupos genéticos e graus de acabamento sobre qualidade da carne de bovinos. Semina: Ciências Agrárias 33:391–402

[CR24] Marcondes MI, Tedeschi LO, Valadares Filho S, de Silva CC, L.F. and, Silva AL, da (2016) Using growth and body composition to determine weight at maturity in Nellore. cattle Anim Prod Sci 56:1121

[CR25] Miguel GZ, Faria MH, Roça RO, Santos CT, Suman SP, Faitarone ABG, Delbem NLC, Girao LVC, Homem JM, Barbosa EK, Su LS, Resende FD, Siqueira GR, Moreira AD, Savian TV (2014) Immunocastration improves carcass traits and beef color attributes in Nellore and Nellore×Aberdeen Angus crossbred animals finished in feedlot. Meat Sci 96:884–891 (Elsevier Ltd) 10.1016/j.meatsci.2013.08.030 24211546

[CR26] NRC (2016) Nutrient Requirements of Beef Cattle, 8th Revised Edition, 8th edn. National Academies, Washington, D.C.

[CR28] Pflanzer SB, de Felício PE (2011) Moisture and fat content, marbling level and color of boneless rib cut from Nellore steers varying in maturity and fatness. Meat Sci 87:7–11 10.1016/j.meatsci.2010.08.009 20855172

[CR29] Prado IN, Rotta PP, Prado RM, do, Visantainer JV, Moletta JL, Perotto D (2008) Carcass Characteristics and Chemical Composition of the Longissimus Muscle of Purunã and 1/2 Purunã vs. 1/2 Canchin Bulls Meat Quality of Bulls Asian-Australasian. J Anim Sci 21:1296–1302

[CR30] Rhoades RD, Sawyer JE, Chung KY, Schell ML, Lunt DK, Smith SB (2007) Effect of dietary energy source on in vitro substrate utilization and insulin sensitivity of muscle and adipose tissues of Angus and Wagyu steers. J Anim Sci 85:1719–1726 10.2527/jas.2006-498 17339414

[CR31] RIISPOA (2017) Regulamento da Inspeção Industrial e Sanitária de Produtos de Origem Animal. Brasília, DF

[CR32] Sheng X, Ni H, Liu Y, Li J, Zhang L, Guo Y (2014) RNA-seq analysis of bovine intramuscular, subcutaneous and perirenal adipose tissues. Mol Biol Rep 41:1631–1637 10.1007/s11033-013-3010-8 24398553

[CR33] Silva LAF, da, Costa AC, da, Soares LK, Borges NC, Ferreira JL, cardoso LL (2009) Orquiectomia em bovinos empregando abraçadeira de náilon na hemostasia preventiva: efeito da estação do ano, método de contenção e técnica cirúrgica. Ciência Anim Brasileira / Brazilian Anim Sci 10:261–270

[CR34] Smith SB, Crouse JD (1984) Relative Contributions of Acetate, Lactate and Glucose to Lipogenesis in Bovine Intramuscular and Subcutaneous Adipose Tissue. J Nutr 114:792–800 10.1093/jn/114.4.792 6716183

[CR35] Snijders SEM, Dillon PG, O’Farrell KJ, Diskin M, Wylie ARG, O’Callaghan D, Rath M, Boland MP (2001) Genetic merit for milk production and reproductive success in dairy cows. Anim Reprod Sci 65:17–31 10.1016/s0378-4320(00)00217-7 11182505

[CR36] Thompson J (2002) Managing meat tenderness Meat Sci 62:295–308 10.1016/s0309-1740(02)00126-2 22061606

[CR37] Urrutia O, Mendizabal JA, Alfonso L, Soret B, Insausti K, Arana A (2020) Adipose tissue modification through feeding strategies and their implication on adipogenesis and adipose tissue metabolism in ruminants. Int J Mol Sci 21 10.3390/ijms21093183 PMC7246642 32365995

[CR38] USDA (2017) United States standards for grades of carcass beef. Washington, DC

[CR39] Warriss PD (2010) Meat science: an introductory text, 2nd edn. Cambridge University Press, Cambridge

[CR40] Webb EC, O’Neill HA (2008) Anim fat paradox meat Qual Meat Sci 80:28–36 10.1016/j.meatsci.2008.05.029 22063167

[CR41] Young OA, Priolo A, Simmons NJ, West J (1999) Effects of rigor attainment temperature on meat blooming and colour on display. Meat Sci 52:47–56 10.1016/s0309-1740(98)00147-8 22062142

