# Supplementary material for: Effect of the genetic merit for backfat thickness on the meat quality of steers
Source: Trop Anim Health Prod. 2026 Jul 20;58(7):421. doi: 10.1007/s11250-026-05241-0 (PMC13385128; doi:10.1007/s11250-026-05241-0)
Supplement: Supplementary file 1 — Supplementary Material 1 [file 11250_2026_5241_MOESM1_ESM.docx]

**Appendix A**

Guaranteed levels of protein-energy supplement used ​​during animal rearing

| Nutritional information | Value |
| --- | --- |
| Moisture (max.) | 120 g/kg |
| Crude protein (min.) | 250 g/kg |
| NNP^1^ (max.) | 170 g/kg |
| Estimated TDN^2^ (min.) | 460 g/kg |
| Calcium (min/max) | 45 - 65 g/kg |
| Cobalt (min.) | 15 mg/kg |
| Copper (min.) | 200 mg/kg |
| Sulfur (min.) | 6,000 mg/kg |
| Fluorine (max.) | 100 mg/kg |
| Phosphor (min.) | 10 g/kg |
| Iodine (min.) | 18 mg/kg |
| Magnesium (min.) | 3,500 mg/kg |
| Manganese (min.) | 185 mg/kg |
| Selenium (min.) | 2 mg/kg |
| Sodium (min.) | 45 g/kg |
| Zinc (min.) | 700 mg/kg |
| Salinomycin sodium (min.) | 85 mg/kg |

Basic composition: Ground corn, soybean meal, wheat bran, citrus pulp pellets, phosphate, difluorized dicalcium, calcium carbonate, sodium chloride (11.66%), ventilated sulfur, magnesium oxide, copper sulfate, cobalt sulfate, manganese sulfate, zinc oxide, calcium iodate, selenite sodium, and salinomycin sodium.

^1^ Non-protein nitrogen equivalent to crude protein.

^2^ Total Digestible Nutrients.

# **Appendix B**

Guaranteed levels of concentrate feed used ​​during animal finishing semiconfined

| Nutritional information | Value |
| --- | --- |
| Moisture (max.) | 117.83 g/kg |
| Crude protein (min.) | 142.18 g/kg |
| NNP^1^ (max.) | 51.00 g/kg |
| Estimated TDN^2^ (min.) | 775.60 g/kg |
| Calcium (min/max) | 7.5 – 13.50 g/kg |
| Cobalt (min.) | 1.95 mg/kg |
| Copper (min.) | 15.00 mg/kg |
| Sulfur (min.) | 1.60 g/kg |
| Fluorine (max.) | 37.50 mg/kg |
| Phosphor (min.) | 3.75 g/kg |
| Iodine (min.) | 3.15 mg/kg |
| Magnesium (min.) | 1.8 g/kg |
| Manganese (min.) | 24.00 mg/kg |
| Selenium (min.) | 0.5 mg/kg |
| Sodium (min.) | 1.5 g/kg |
| Zinc (min.) | 60 mg/kg |
| Monensin sodium (min.) | 19.95 mg/kg |
| Salinomycin sodium (min.) | 25.05 mg/kg |

Basic composition: Ground corn, soybean meal, wheat bran, citrus pulp pellets, phosphate, difluorized dicalcium, calcium carbonate, sodium chloride, ventilated sulfur, magnesium oxide, copper sulfate, cobalt sulfate, manganese sulfate, zinc oxide, calcium iodate, selenite sodium, urea, monensin sodium and salinomycin sodium. Obtained by mixing commercial mineral core with energy bran (ground corn) in a proportion of 15:85, respectively.

^1^ Non-protein nitrogen equivalent to crude protein.

^2^ Total Digestible Nutrients.

# **Appendix C**

# Formulation and composition of the feedlot diet

| Ingredient | g/kg DM | | | | | |
| --- | --- | --- | --- | --- | --- | --- |
| Sorghum silage | 500.0 | | | | | |
| Ground corn grain | 240.0 | | | | | |
| Soy bark | 149.0 | | | | | |
| Soybean meal | 80.0 | | | | | |
| Urea | 11.0 | | | | | |
| Mineral core ^1^ | 20.0 | | | | | |
| Composition (g/kg) | TMR^2^ | Silage | Concentrate | Corn | Soybean meal | Soy bark |
| Dry matter | 483.40 | 330.18 | 892.72 | 882.55 | 893.28 | 868.62 |
| Crude protein | 122.18 | 49.71 | 223.91 | 87.86 | 486.96 | 139.35 |
| Mineral matter | 61.00 | 53.26 | 112.15 | 25.61 | 74.65 | 40.12 |
| Neutral detergent fiber | 473.31 | 573.04 | 322.19 | 118.66 | 141.38 | 631.88 |
| Acid detergent fiber | 311.99 | 363.42 | 237.65 | 38.25 | 95.98 | 503.04 |
| Total digestible nutrients^3^ | 640.48 | 598.88 | 703.52 | 788.41 | 778.93 | 574.34 |
| Digestibility (%) | | | | | | |
| IVOMD^4^ | 64.25 | 53.85 | 74.96 | 73.34 | 68.18 | 72.83 |
| Energy (Mcal/kg of DM) | | | | | | |
| Digestible energy^5^ | 2.82 | 2.64 | 3.10 | 3.47 | 3.43 | 2.53 |
| Metabolizable energy^5^ | 2.31 | 2.16 | 2.54 | 2.84 | 2.81 | 2.07 |

^1^ Minimum guaranteed levels per kg: calcium 250g (maximum 290 g); phosphorus 22 g; sodium 40 g; magnesium 15 g; sulfur 30 g; fluorine 220 mg; zinc 2200 mg; copper 500 mg; manganese 800 mg; cobalt 35 mg; iodine 45 mg; selenium 20 mg; vitamin A 60000 IU; vitamin D 20000 IU; vitamin E 400 IU; sodium monensin 1000 mg; *Saccharomyces cerevisiae* 1.0 x 10^7^ CFU; virginiamycin 1000 mg.

^2^ Total mixed ration.

^3^ Calculated according to Cappelle *et al*. (2001).

^4^ *In vitro* organic matter digestibility.

^5^ Estimated according to NRC (1996), considering 1 g TDN = 0.0044 Mcal of digestible energy (DE) and 1 Mcal of DE = 0.82 Mcal of metabolizable energy.
